# Association Between Zinc Levels and the Impact of Its Deficiency on Idiopathic Male Infertility: An Up-to-Date Review

**DOI:** 10.3390/antiox14020165

**Published:** 2025-01-29

**Authors:** Nebojša Zečević, Aleksandra Veselinović, Milan Perović, Aleksandar Stojsavljević

**Affiliations:** 1 Clinic for Gynecology and Obstetrics “Narodni Front”, Kraljice Natalije 62, 11000 Belgrade, Serbia; nebojsa.zecevic1@gmail.com (N.Z.); perovicmilan@hotmail.com (M.P.); 2 Faculty of Medicine, University of Belgrade, Doktora Subotića 8, 11000 Belgrade, Serbia; 3 Special Hospital Belgrade, Human Reproduction Center, Antifašističke borbe 2a, 11000 Belgrade, Serbia; 4 Cognitive Neuroscience Department, Research and Developmental Institute “Life Activities Advancement Institute”, 11000 Belgrade, Serbia; a.veselinovic@add-for-life.com; 5 Department of Speech, Language and Hearing Sciences, Institute for Experimental Phonetics and Speech Pathology, 11000 Belgrade, Serbia; 6 Innovative Centre of the Faculty of Chemistry, University of Belgrade, Studentski trg 12-16, 11000 Belgrade, Serbia

**Keywords:** infertility, zinc, deficiency, sperm quality, supplementation

## Abstract

Background: Zinc (Zn) is an essential metal that plays a critical role in normal testicular development, spermatogenesis, prevention of sperm degradation, and overall male fertility. This review aims to offer a comprehensive and current overview of seminal plasma Zn levels in fertile men worldwide. It also aims to compare Zn levels in seminal plasma and blood (serum/plasma) between infertile men (cases) and fertile men (controls), examine the impact of Zn on sperm quality and the reproductive hormone, and highlight the effects of Zn supplementation therapy in male infertility. Methods: To achieve these goals, peer-reviewed studies from 2000 to 2024 were interrogated with regard to strict inclusion/exclusion criteria and were then thoroughly reviewed and analyzed. Results: Our findings indicate that maintaining optimal seminal plasma Zn levels is crucial, as low Zn levels are linked to impaired spermatogenesis and male infertility, while high Zn levels can cause oxidative stress and other changes that contribute to infertility. Seminal plasma Zn levels from 100 to 200 mg/L among fertile men worldwide can be roughly considered safe. Comparative analysis showed that a greater number of studies reported lower levels of seminal Zn in cases than in controls. Research into the impact of Zn levels in seminal plasma has shown that, although the results are not yet conclusive, altered (non-normal) Zn levels could influence semen parameters—particularly motility, morphology, and sperm count—and the level of the reproductive hormone, testosterone. Zinc-deficient infertile men could benefit from supplement therapy. Conclusions: Assessment of seminal plasma Zn levels in infertile men could provide valuable information and aid in diagnosis and treatment planning.

## 1. Male Infertility

Infertility is defined as the failure to conceive after a year or more of regular sexual intercourse without the use of contraception and is the result of impaired reproductive ability, either individually or with a partner [1]. Infertility has become a serious health and socio-economic–psychological problem globally [2]. Approximately 15% of couples worldwide experience infertility, equivalent to 48.5 million couples [3]. However, male partners contribute to 20–30% of all infertility cases [4]. Across the globe, infertility rates are higher in Eastern Europe, North Africa, and the Middle East [5]. Declining male fertility is a common health problem worldwide due to the deteriorating quality of sperm over the years; moreover, a decrease in sperm concentration (−0.64 million/mL per year) was recorded from 1973 to 2011 [6]. Huang et al.’s (2017) longitudinal study from 2001 to 2015 showed noticeable decreasing trends in sperm quality: sperm concentrations dropped from 68 × 10^6^/mL to 47 × 10^6^/mL, progressively motile sperm count dropped from 34 × 10^6^ to 21 × 10^6^, and the proportion of sperm with normal morphology dropped from 31.8 to 10.8% [7]. The main exogenous factors leading to male infertility are lifestyle changes, poor diet, increased obesity rates, genetic predispositions, endocrine imbalances, and exposure to environmental toxins—heavy metals, pesticides, dioxins, and solvents, known as endocrine-disrupting compounds (EDCs) [8,9,10].

Human semen is a rich mixture that plays a pivotal role in reproduction. It consists of seminal plasma (95%) and testicular secretions with sperm cells (5%) [11]. Seminal plasma is the liquid part of semen and carries the sperm cells as they pass through the male reproductive tract during ejaculation. Seminal plasma is involved in the regulation of capacitation and in the protection and maturation of sperm, and it possesses antimicrobial properties, among other functions [12,13]. In elucidating the pathogenesis of male infertility, the scientific community is increasingly favoring markers in semen or seminal plasma than in serum/plasma, urine, and other body fluids [14,15].

A basic seminogram (semen analysis) is considered essential for assessing male fertility. Although semen analysis is widely used, it lacks the precision to reliably differentiate between infertile and fertile men, serving primarily to classify men as subfertile [16,17]. The World Health Organization (WHO) recommends performing two or three seminograms at different time periods to obtain reliable information about the patient’s semen parameters. However, determining conventional semen parameters is not satisfactory to assess male fertility since 20–30% of cases have idiopathic (unexplained) male infertility [18]. Gaining insight into the genetic aspect of idiopathic male infertility is also important in managing reproductive challenges. Genetics could affect various physiological processes, such as hormonal balance, spermatogenesis, and sperm quality [19]. Chromosomal abnormalities (translocations, Klinefelter syndrome) and Y chromosome microdeletions are responsible for about 5% of male infertility [20,21]. Single gene mutations could be expected in men with idiopathic male infertility and could include genes involved in spermatogenesis (*DAZ, USP9Y*, and *RBMY*) [22] or in sperm function (CATSPER) [23]. Males who carry the rs4804490 single nucleotide polymorphism in the *DNMT1* could be at higher risk of idiopathic infertility [24]. It is estimated that more than 2000 genes (housekeeping and germ cell-specific genes) are involved in spermatogenesis [25]. In addition to genetics, environmental factors markedly influence male infertility [9]. All of this points to the need for new biomarkers that would clearly distinguish infertile from fertile men [26].

According to the WHO, normozoospermia is a positive seminogram finding with the following data: sperm count > 15 million/mL, total motility 40%, of which 30% have active motility, and >4% normal morphology (lower reference limit, LRL). The terminology referring to altered seminogram findings includes oligozoospermia (the total sperm count is less than the LRL); asthenozoospermia (the percentage of progressively motile sperm cells is below the LRL); teratozoospermia (the percentage of morphologically normal sperm cells is less than the LRL); azoospermia (absence of sperm cells in the ejaculate); cryptozoospermia (spermatozoa absent in fresh preparations but visible in centrifuged pellet); and combined seminogram types, such as oligoasthenoteratozoospermia [25,27].

This review aims to comprehensively provide in-depth and up-to-date information on seminal plasma Zn levels among fertile men worldwide, compare seminal plasma and blood serum/plasma Zn levels among infertile men (cases) and fertile men (controls), shed light on the influence of Zn on sperm quality and reproductive hormones, and highlight the effects of Zn supplementation therapy in male infertility. To achieve these goals, peer-reviewed studies from 2000 to 2024 were interrogated with regard to strict inclusion/exclusion criteria (see below) and were then thoroughly reviewed and analyzed.

## 2. The Role of Zinc and Its Altered Levels on the Male Reproductive System

Zinc is an essential trace metal with antioxidant and anti-inflammatory roles in the human body [28]. Due to its redox-inactive nature, Zn does not participate in redox reactions (Zn^2+^). Zinc is a cofactor of DNA-binding proteins with Zn fingers, copper/zinc superoxide dismutase (Cu/Zn SOD), and over 300 other proteins/enzymes, including those involved in DNA damage repair, transcription, and translation processes [29,30]. Zinc has three functions in enzymes: (1) catalytic function (directly participates in the enzyme’s catalytic activity, enabling biochemical reactions), (2) coactive function (catalytic Zn enhances or moderates the enzyme’s activity), and (3) structural function (Zn helps in maintaining the stability of the enzyme’s quaternary structure) [31].

Among all trace elements in the body, Zn is second in abundance to iron (Fe) [32]. The adult body contains 2–3 g of Zn [33]. Specifically, 49.5% of Zn is found in muscles, 36.7% in bones, 7.6% in skin, and 6.2% in all other organs [34]. The biological half-life of Zn is about 280 days. Zinc levels in the bloodstream are low [35]. At the cellular level, 30–40% of Zn is localized in the nucleus, 50% in the cytosol, and the remaining portion is associated with membranes [36].

Zinc is mainly found in animal proteins (meat and milk), fish and seafood (e.g., oysters), legumes, certain mushrooms, nuts, and fortified cereals [36]. The average Zn intake for men is about 14 mg/day and 9 mg/day for women [31]. Net intestinal Zn intake is regulated and varies from 20 to 50% of dietary intake; at an intake of 12.2 mg Zn/day, fractional absorption is 26%, but at a very low intake of 0.23 mg Zn/day, this increased to 100% [31]. Interaction with other dietary constituents, such as phytic acid, calcium, and Fe, considerably reduces the net absorption of Zn [37]. Therefore, strict vegetarians may need up to 50% more Zn per day due to the high phytic acid and fiber contents in their diet [38]. Absorbed Zn is transported to the liver via portal circulation, where it is actively incorporated into metalloenzymes and plasma proteins, such as albumin and α2-macroglobulin [39]. Zinc circulating in blood plasma makes up about 0.1% of the total body stores [40]. The clinical guideline reference interval for serum Zn is 800 to 1300 μg/L [31]. However, numerous studies have warned that this range should be taken with caution due to considerable differences in Zn levels among populations in different countries across the globe [41,42].

Zinc is crucial for normal testicular development, spermatogenesis, prevention of sperm degradation, and, ultimately, reproductive health [43]. Zinc content accumulates in germ cells over time and increases in the testes during spermatogenesis. The prostate, testicles, and epididymis are rich in Zn. Most of the Zn in seminal plasma comes from the prostate [36]. The prostate contains 150 µg/g of Zn, which is approximately three times more than in any other soft tissue. Therefore, prostatic fluid is very rich in this metal, with approximately 500 µg Zn/mL [44]. Additionally, Zn levels in ejaculate are 85- to 90-fold higher than in blood plasma [45]. Zinc affects lipid flexibility and sperm membrane stabilization and regulates capacitation and the acrosome reaction (key processes for conception and embryo implantation) [46]. At the end of spermatogenesis, Zn is most abundant in the tail of mature sperm and is closely associated with sulfhydryl groups and disulfide linkages; thus, it is pivotal for sperm motility [36]. Zinc acts as a regulator of disulfide cross-links in the sperm nucleus by forming a precise number of HS-Zn-SH structures [47]. In addition, Zn competes with redox-active metals (primarily Fe and Cu) for specific proteins and compounds on cell membranes, displacing these trace metals and making them more available for binding to their transport proteins [48]. Zinc ions also have an antimicrobial role in seminal plasma [49].

Zinc plays an essential role in maintaining epithelial integrity (vital for preserving the lining of the reproductive organs) [50] and regulating the processes of capacitation and the acrosome reaction (both of which are essential for successful fertilization) [51], as well as in nuclear chromatin decondensation after fertilization [52]. Conversely, low Zn levels disrupt spermatogenesis, contribute to sperm abnormalities, and negatively impact serum testosterone levels [36]. However, while numerous studies demonstrate a link between seminal plasma Zn concentration and sperm physiology, it cannot be definitively concluded that seminal Zn deficiency directly causes infertility [53].

High or low levels of Zn in the body impair male reproductive functions, which is why maintaining homeostasis of this element is crucial [53]. According to the WHO, Zn deficiency affects about one-third of the world’s population [54]. Dietary Zn deficiency (<5 mg/g) impairs male reproductive health and could be a critical risk factor for idiopathic male infertility [36]. Thus, Zn deficiency is associated with gonadal dysfunction, reduced testicular weight/volume, Leydig cell damage, shrinkage of the seminiferous tubules, hypogonadism, sex hormone deficiency, impaired spermatogenesis, oxidative stress, inflammation, and apoptosis [55,56]. In a large cross-sectional and retrospective study conducted by Hibi et al. (2020) [57] in Japanese men (mean age = 36 years), reference serum Zn levels (800–1300 μg/L) were observed in 1069 (53.2%) infertile men, subclinical deficiency (≥600 to <800 μg/L) in 845 (42.0%) men, and deficiency (<600 μg/L) in 79 (3.9%) men, while high serum Zn levels (>1310 μg/L) were found in only 17 men (0.9%) [57]. On the other hand, excess Zn can induce oxidative stress in men, inhibit capacitation and the acrosome reaction, and produce other altered states. Following the findings that alpha-lipoic acid improves fertility in women [58,59], lipoic acid has also been used in men facing subfertility/infertility. Interestingly, Zn-induced oxidative stress in male reproductive organs was decreased by lipoic acid treatment [60,61]. Therefore, lipoic acid supplementation could be used in those men with high levels of Zn that harm male reproductive functions.

### 2.1. Comparative Analysis of Zn Levels in Seminal Plasma Worldwide

**Figure 1:**
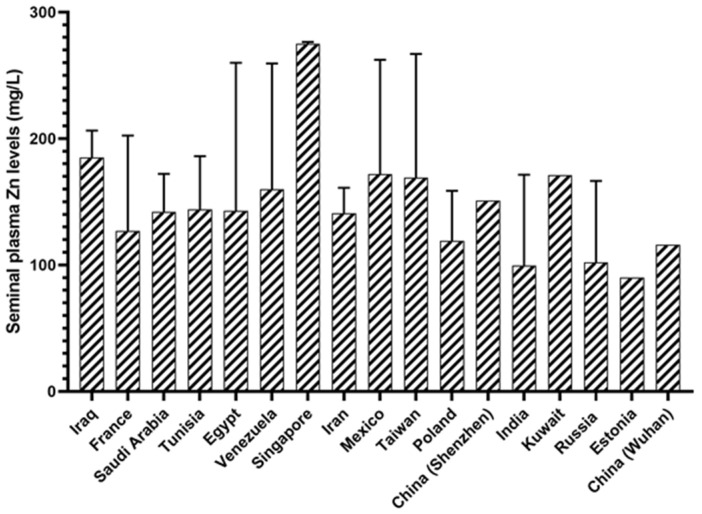
Zinc levels in the seminal plasma of fertile men in countries or regions worldwide. Data are presented as mean and standard deviation or as median (mg/L). The figure was drawn using GraphPad Prism (v.5.0, CA, USA).

According to the literature data, Zn levels in the seminal plasma of fertile men are partially available among populations worldwide. In this review analysis, we enrolled those studies that adhered to the 2010 WHO laboratory manual for the examination and processing of human semen. In addition, all studies that did not specify exclusion criteria for fertile participants (primarily ejaculatory obstruction, testicular failure, inadequate sperm concentration or absence, aberrant sperm morphology and motility, age, obesity (BMI ≥ 30)) were not considered in this review. The results for seminal plasma Zn levels of normozoospermic men worldwide are shown in Figure 1. Generally, with the exception of Singapore, Zn levels in seminal plasma ranging roughly from 100 to 200 mg/L can be considered safe for male fertility. The authors of this review urge researchers to determine Zn levels in the seminal plasma of subjects in as many countries as possible, even in regions of the same country, to draw more reliable (statistical) conclusions about seminal plasma Zn levels of fertile men across the world.

### 2.2. Comparative Analysis of Seminal and Blood Zn Levels Between Cases and Controls

This section includes only studies that offered comprehensive details about participants, along with Zn levels in seminal plasma and/or blood. Thus, studies without sufficient numerical data, studies before 2000, studies where the age of cases and controls did not match, studies where comorbidities and mineral/supplement therapy were not excluded, and studies in a language other than English were not considered in our review analysis.

According to our review analysis, the largest number of studies reported significantly lower Zn levels in the seminal plasma and blood of infertile men than in fertile men. Thus, Aljaser et al. (2021) found significantly lower seminal plasma Zn levels in 30 asthenozoospermic men (120 ± 30.8 mg/L) than in 40 control men (142 ± 30.2 mg/L) [62]. Atig et al. (2012) found significantly lower seminal Zn levels in all three groups of infertile men (74 asthenozoospermic (122 ± 34.7 mg/L), 56 oligozoospermic (121 ± 25.3 mg/L), and 60 teratozoospermic men (126 ± 24.8 mg/L)) than in 60 normozoospermic men (144 ± 42.1 mg/L) [63]. Chia et al. (2000) compared Zn levels in seminal plasma and blood between 107 infertile men and 103 fertile men. They found significantly lower seminal plasma Zn levels in cases (geometric mean level = 184 mg/L) than in controls (geometric mean level = 275 mg/L). No significant differences were found between blood Zn levels between infertile and fertile men (Chia et al. 2000) [64]. Chyra-Jach et al. (2020) compared seminal plasma Zn levels between 152 oligozoospermic, 142 asthenozoospermic, and 90 oligoasthenozoospermic men with 103 normozoospermic men and found significantly lower seminal plasma Zn levels in men with asthenozoospermia than in normozoospermic men. The main drawback of this study is that the authors presented the data graphically, instead of numerically [65]. Colagar et al. (2009) compared seminal plasma Zn levels in 17 fertile smokers and 19 fertile nonsmokers with 15 infertile smokers and 21 infertile nonsmokers. They found that infertile smokers (80.7 ± 26.5 mg/L) and infertile nonsmokers (103 ± 29.8 mg/L) had significantly lower seminal plasma Zn levels than fertile smokers (124 ± 26.4 mg/L) and fertile nonsmokers (141 ± 20.1 mg/L). They also found lower seminal plasma Zn levels in smokers than in nonsmokers, with no statistical differences [50]. Saleem et al. (2021) compared seminal Zn levels between 50 fertile men with 50 non-obstructive azoospermic men and 50 men with idiopathic oligoasthenoteratozoospermia (iOAT) [66]. They found that azoospermic men had significantly lower seminal Zn levels than fertile men and men with iOAT.

Interestingly, Türk et al. (2014) compared 29 men with severe prostatitis (>10^6^ white blood cells in prostate secretion), 31 men with severe leukocytozoospermia (>10^6^ white blood cells in semen), 24 men with mild inflammation (0.2–1.0 mol/L white blood cells in semen or prostate secretion), 32 men with non-inflammatory oligozoospermia, and 27 healthy men (control group). They found that male partners of all infertile couples/groups had lower seminal plasma Zn levels than the control group [67]. Hibi et al. (2022) showed that serum Zn levels in normozoospermic men were significantly higher than in azoospermic men (non-obstructive and obstructive) and cryptozoospermic men. Also, serum Zn levels were lower in non-obstructive azoospermicmen than in oligozoospermic and asthenozoospermic men [57]. Vashisht et al. (2021) enrolled 50 infertile and 50 age-matched fertile men. They split 50 infertile men into subgroups and found significantly lower seminal plasma Zn levels in 13 asthenozoospermic men (62.7 ± 9.40 mg/L), 16 non-obstructive azoospermic men (63.9 ± 8.33 mg/L), and 11 oligozoospermic men (74.7 ± 10.1 mg/L) than in fertile men (134 ± 4.85 mg/L) [68]. Akinloye et al. (2011) found that the Cu/Zn ratio in serum and seminal plasma was significantly lower in the groups with altered seminograms than in the group of men with normal seminograms [69].

Sun et al. (2017) included eight studies (total number of participants = 1029) in their meta-analysis and found significantly lower seminal plasma Zn levels in infertile men than in fertile men [70]. Zhao et al. (2016) also conducted a meta-analysis enrolling 2600 cases and 867 controls, reporting significantly lower seminal plasma Zn levels in infertile men than in fertile men. Pooling data from six studies, they also found that Zn supplementation significantly elevated sperm volume, sperm motility, and the percentage of normal sperm morphology [45].

Several studies found no statistically significant differences in Zn levels in seminal plasma and blood between cases and controls, although the authors generally noted lower Zn levels in cases than in controls. Abdul-Rasheed (2009) compared seminal plasma Zn levels in 39 normozoospermic/control men with 22 azoospermic men, 32 oligozoospermic men, and 6 asthenozoospermic men. No significant differences were found for seminal Zn levels between the control group (185 ± 21.3 mg/L), azoospermic men (179 ± 18.6 mg/L), oligozoospermic men (182 ± 23.4 mg/L), and asthenozoospermic men (195 ± 13.0 mg/L). However, the author also determined high-molecular-weight zinc-binding protein (HMW-Zn) (a parameter of seminal plasma chelating capacity and a measure of Zn bioavailability) and reported significantly lower values among azoospermic men (8.54 ± 1.89%), oligozoospermic men (6.80 ± 1.11%), and asthenozoospermic men (6.38 ± 1.43%) compared to the sperm fraction/percentage for control men (12.6 ± 1.26%). Based on numerical data, it can be concluded that seminal HMW-Zn values were lowest in asthenozoospermia (about two-folds lower than in the control group). The author also reported a significant positive correlation between HMW-Zn and the percentage of progressive motility [71]. Interestingly, Camejo et al. (2011) compared the seminal plasma Zn levels in 44 men with normozoospermia and 67 men with varicocele (grades II + III) and found no significant differences between the groups [72]. Jeng et al. (2015) compared two groups <40 vs. >40 years), but men over 40 years of age had significantly lower levels of Zn^2+^ ions in semen than of participants based on differences in sperm concentrations (<15 × 10^6^/mL vs. ≥15 × 10^6^/mL) [73]. They discovered that there were no significant differences in Zn levels in seminal plasma and urine between the two groups. Zafar et al. (2015) found no significant differences between the levels of Zn in seminal plasma of 25 fertile men, 25 oligozoospermic men, and 25 azoospermic men (150 ± 44.8 mg/L, 111 ± 78.3 mg/L and 139 ± 65.2 mg/L, respectively) [74]. Chen et al. (2024) showed no significant differences in Zn^2+^ in semen between four age groups (≤30 vs. 31–35 vs. 36–40 other three age groups. The authors found no statistical difference in the total amount of Zn^2+^ per ejaculation or Zn^2+^ levels in semen in 374 normozoospermic men, 525 oligoasthenozoospermic, and 65 azoospermic men [75].

While the existing literature suggests lower Zn levels in the blood (serum/plasma) and seminal plasma of infertile men compared to fertile men, we emphasize the need for further research to confirm this association with greater reliability. To achieve this, the authors of this review stress that, in the future, it is necessary to increase the number of fertile and infertile men, classify a larger number of infertile men based on semen analysis, and determine Zn levels in seminal plasma and blood of the same individuals.

### 2.3. Impact of Zinc on Seminogram Findings

The literature data collected from 2000 to 2024 on the influence of Zn levels in seminal plasma, semen, and/or blood on sperm parameters are summarized in Table 1, first as case–control studies and second as cross-sectional studies. The data consistently indicate that Zn positively impacts semen parameters, significantly improving sperm motility, morphology, and sperm count. However, further studies with well-defined methodologies are required to more reliably (statistically) establish the association between Zn and specific seminogram parameters.

### 2.4. Impact of Zinc on the Reproductive Hormone, Testosterone

Zinc plays a key role in the biosynthesis, storage, and secretion of male sex hormones, primarily testosterone [68,81]. Testosterone is an androgen produced by Leydig cells and has regulatory effects on spermatogenesis [69,82]. Zinc is predominantly present in Leydig cells, late type B spermatogonia, and spermatids. In addition, Zn is a cofactor of 5α-reductase, which is the crucial enzyme for the transformation of testosterone into its biologically active form, dihydrotestosterone [36].

The literature data imply that low Zn levels have been associated with low testosterone levels. An older clinical study with 11 volunteers conducted by Hunt et al. (1992) on different Zn dietary regimens for 28 days showed that serum testosterone levels, seminal volume, and total seminal Zn loss per ejaculate were sensitive to short-term Zn depletion in young men [70,83]. Egwurugwu et al. (2013) [71,84] and Omu et al. (2015) [72,85] demonstrated, in mice, that Zn deficiency can lead to Leydig cell apoptosis, failure of steroidogenesis, decreased testosterone and progesterone levels, and increased luteinizing hormone (LH) and follicle-stimulating hormone [84,86]. Akinloye et al. (2011) found that serum and seminal testosterone levels were significantly lower in men with normozoospermia than in oligo- and azoospermic men, and the differences were more pronounced in azoospermic men than in oligospermic men. In addition, serum Zn levels were significantly and positively correlated with seminal testosterone levels, while seminal Zn levels were significantly and positively correlated with serum LH and FSH levels [57,69]. Kothari et al. (2016) reported a positive association between seminal plasma Zn levels, testicular steroidogenesis, and serum-free testosterone [73,87]. Saleem et al. (2021) reported that men with idiopathic oligoasthenoteratozoospermia had a positive and significant correlation between serum testosterone levels and seminal plasma Zn levels [54,66]. On the other hand, Osadchuk et al. (2021) reported a negative correlation between serum Zn levels and serum testosterone and estradiol levels. They also separated 626 men according to the WHO reference limits (≥2.4 μmol Zn/ejaculate as normal group and <2.4 μmol Zn/ejaculate as deficit). They found that testosterone levels were 7.1% lower in the group with seminal Zn deficiency than in the group with normal seminal Zn [67,80]. However, Hibi et al. (2022) showed that serum Zn levels were not correlated with sperm concentration, sperm motility, LH, FSH, and testosterone. Further studies are needed to more clearly highlight the role of Zn on testosterone levels in seminal plasma and blood [48,57].

According to our extensive search, Shafiei et al. (2011), Jalali et al. (2010), Brilla and Conte (2000), and Koehler et al. (2009) have examined the effects of Zn supplementation on blood testosterone levels [86,88,89,90]. In summary, the first two studies reported significantly higher total testosterone levels after treatment than before treatment, while the other two studies did not find a significant improvement in total testosterone levels after treatment. Details are provided in Table 2. The contradicting findings stated need to be addressed promptly in the future.

## 3. Impact of Zinc Therapy on Male Infertility

In this section, we reviewed studies from 2000 to 2024 that addressed the effects of Zn supplements on sperm parameters. The most commonly used Zn supplement was zinc sulfate (ZnSO_4_), given orally in various doses (20–440 mg) for different periods of time. Chronological details of the included studies are given below.

Wong et al. (2002) enrolled 203 subfertile men and 108 fertile men from the Netherlands. The participants were randomly assigned to eight groups, i.e., six intervention groups and two control groups. Two intervention groups (one composed of 23 infertile men and the other of 48 fertile men) received oral Zn supplementation (66 mg/day, 6 months). They were reassessed at the end of the treatment period and their results were compared with baseline values. The authors reported a significant improvement in sperm count in infertile men, while sperm count was also higher in fertile men but without statistical significance [78,91]. In the same study, Wong et al. (2002) enrolled 103 subfertile and 108 fertile men and conducted a double-blind, randomized, placebo-controlled trial on the effects of the same dose of Zn for the same duration, but with the addition of folic acid (FA) (5 mg/day). The authors’ results are almost in line with the previous findings: sperm count elevated from about 7.5 × 10^6^/mL before to 12 × 10^6^/mL after treatment (almost 74% increase) for both subfertile and fertile men, while significant differences in other semen parameters were not found [78,91].

Ebisch et al. (2003) conducted a double-blind, placebo-controlled intervention study in which they enrolled 113 fertile and 77 subfertile men from the Netherlands. Participants were divided into four groups (placebo, group receiving 66 mg Zn/day, group receiving 5 mg FA daily, and group receiving 66 mg/day Zn + 5 mg FA/day). The duration of therapy was 26 weeks for all groups. They found a significant increase in sperm count only in the group receiving Zn + FA [79,92]. Three years later, Ebisch et al. (2006) enrolled 49 fertile and 40 subfertile men, divided into four groups: fertile and infertile (both 132 mg of ZnSO_4_ for 6 months), while the other two groups were placebo. They discovered that subfertile men had a considerable increase in sperm count after treatment, which was not observed in fertile men, demonstrating the treatment’s focused efficiency [80,93].

Hadwan et al. (2012) enrolled 37 subfertile men (asthenozoospermic) and 37 fertile (normozoospermic) men from Iraq. The subfertile group was treated with 220 mg of ZnSO_4_ twice daily for three months, while the fertile group was assigned as a control. They reported significant improvement in sperm volume, motility, and morphology for the asthenozoospermic men compared to the control group [81,94]. Two years later, Hadwan et al. (2014) applied the same methodology, dose, timing, and outcome evaluation as in the previous study, but for a slightly larger number of participants (60 subfertile + 60 fertile men). They obtained the same findings (numerically increased sperm volume and motility and improved morphology in the subfertile men), also in addition to a significant improvement in sperm count in cases than in controls [82,95].

Raigani et al. (2014) enrolled 85 men with oligoasthenozoospermia from Iran. They were divided into four groups (10 mg of FA, 10 mg of FA + 440 mg of Zn, 440 mg of Zn, and placebo). There were no statistically significant differences between the groups for any sperm parameter [83,96].

Fatima et al. (2015) included 75 men with oligoasthenozoospermia and divided them into group A (n = 38, 20 mg ZnSO_4_ twice daily for 12 weeks) and group B (n = 37, placebo/glucose tablets). They found that serum Zn levels in group A showed a significant improvement after treatment (+198 mmol/L). Similarly, seminal plasma Zn levels were also improved after supplementation (+942 mmol/L). They also found significant positive correlations after Zn supplementation in sperm count, motility and rapid linear motility, and morphology in group A [84,97].

Schisterman et al. (2020) conducted a multicenter, double-blind, randomized, placebo-controlled clinical trial and enrolled two groups, one receiving 30 mg of elemental Zn and 5 mg of folic acid daily for 6 months (n = 1185) and one placebo (n = 1185). They found no significant differences in live birth rates of couples or in sperm parameters (sperm count, motility, volume, morphology, and total number of motile sperm) between groups [85,98].

Li et al. (2023) conducted a meta-analysis of eight studies that examined the effects of a combination of either ZnSO_4_ and FA or ZnSO_4_ alone or FA alone. The authors conducted a statistical analysis combining all the studies and found no significant improvements in sperm parameters in the treated groups compared to the untreated groups [86,99].

Considering the findings of the previously mentioned studies highlighting the combination of ZnSO₄ and other antioxidants like FA, it is also important to emphasize the antioxidant properties of the Zn and inositol combination. Studies on male reproduction have often predominantly relied on in vivo animal models and female reproduction models, a trend also observed in research on Zn and Vitamin D. Following the findings of the beneficial role of inositols and Vitamin D supplementation on female reproduction [100,101,102,103], data regarding the same issue in men emphasize that inositol supplementation in combination with Zn concomitantly ameliorates sperm characteristics such as concentration, morphology, and motility [104].

Overall, the existing literature modestly suggests that Zn supplementation alone is effective, while the combination of Zn with FA appears less beneficial in improving sperm quality. However, the variability in study designs, including differences in the number of participants, Zn doses, and treatment durations, makes it challenging to draw reliable conclusions. It appears that only infertile men with Zn deficiency can benefit from Zn supplementation. This is particularly noteworthy since Zn in sulfate, citrate, gluconate, and other forms has been widely used in clinical practice for years to treat male infertility. Thus, new, high-quality randomized controlled clinical trials are urgently needed to confirm or refute the efficacy of Zn supplementation therapies, alone or in combination with FA for infertile men.

## 4. Conclusions and Further Directions

In summary, we found that seminal plasma Zn levels should be maintained at optimal levels: low Zn levels are associated with impaired spermatogenesis and overall male infertility, while high Zn levels lead to oxidative stress and other alterations that result in male infertility. Seminal plasma Zn levels from 100 to 200 mg/L among fertile men worldwide can roughly (without statistical analysis) be deemed safe. This comparative analysis shows that the largest number of studies support there being markedly lower Zn levels in the seminal plasma of infertile than in fertile men. Studies on the impact of seminal plasma Zn levels have shown that, although the results are not yet conclusive, altered Zn levels could affect semen parameters, primarily motility, morphology, and sperm count, but also testosterone. Additionally, Zn-deficient infertile men could benefit from supplement therapy. Assessment of seminal plasma Zn levels in infertile men can provide valuable insights and aid in diagnosis and treatment planning. Details remain to be explored on whether Zn levels in seminal plasma and/or the serum of infertile men can serve as biomarkers in clinical practice. The authors of this review strongly suggest the need for larger homogeneous cohorts, uniformly randomized double-blind trials, and, consequently, meta-studies to further highlight the overall roles of Zn in male idiopathic infertility. Yet, we believe that the information provided in this review will be of great importance for deepening knowledge about the impact of Zn on male infertility.

## Figures and Tables

**Table 1:** The literature data on the influences of Zn on seminogram findings (retrieved from 2000 to 2024).

References	Study Design	Country	Sample Size	Age (Years)	Clinical Sample	Analytical Technique	Simplified Main Findings
Akinloye et al. (2011) [69]	Case–control	Nigeria	60 infertile men (40 oligozoospermic + 20 azoospermic) and 40 normozoospermic men	20–55	Serum, seminal plasma	AAS	A significant negative correlation between serum Zn level and sperm counts. A significant negative correlation between seminal Zn level and sperm viability. All data refer to cases.
Aljaser et al. (2021) [62]	Case–control	Saudi Arabia	40 normozoospermic and 30 asthenozoospermic men	36.8 ± 4.91	Seminal plasma	AAS	Significant positive correlations between seminal Zn levels with sperm motility and normal morphology. All data refer to cases.
Atig et al. (2012) [63]	Case–control	Tunisia	60 normozoospermic men and 190 infertile men (74 asthenozoospermic, 56 oligozoospermic, and 60 teratozoospermic)	22–50	Seminal plasma	AAS	Significant positive correlations between seminal Zn levels with sperm motility and sperm count. All data refer to cases.
Camejo et al. (2011) [72]	Case–control	Venezuela	44 normozoospermic men and 67 men with varicocele	34.3 ± 6.4	Seminal plasma	TXRF	A significant positive correlation between seminal Zn level and sperm count. All data refer to cases.
Chia et al. (2000) [64]	Case–control	Singapore	107 infertile and 103 fertile men	34.2 ± 4.30	Seminal plasma, blood	AAS	Significant positive correlations between seminal Zn levels with sperm density, motility, and viability. All data refer to cases.
Chyra-Jach et al. (2020) [65]	Case–control	Poland	346 infertile men (152 oligozoospermic, 142 asthenozoospermic, and 90 oligoasthenozoospermic), 103 fertile men	34 ± 5	Seminal plasma	AAS	Significant positive correlations between seminal Zn levels with sperm count and morphology. All data refer to cases.
Colagar et al. (2009) [50]	Case–control	Iran	72 men grouped as fertile nonsmokers (n = 19), fertile smokers (n = 17), infertile nonsmokers (n = 21), and infertile smokers (n = 15)	31.3 ± 4.05	Seminal plasma	AAS	Significant positive correlations between seminal Zn levels with sperm count and normal morphology. All data refer to cases.
Khan et al. (2011) [76]	Case–control	Pakistan	1521 infertile and 97 fertile men	34.6 ± 0.26	Semen	Spectrophotometry	Significant positive correlations between seminal Zn levels with sperm count and motility. All data refer to cases.
Mankad et al. (2006) [77]	Case–control	India	20 infertile and 55 fertile men	30.3 ± 5.70	Seminal plasma	AAS	A significant positive correlation between seminal Zn level and sperm count. All data refer to cases.
Saleem et al. (2021) [66]	Case–control	Egypt	100 infertile men (50 men with non-obstructive azoospermia + 50 men with iOAT) and 50 fertile men	34.3 ± 4.17	Seminal plasma	Spectrophotometry	A significant positive correlation between seminal Zn level and seminal volume. All data refer to cases.
Wong et al. (2001) [78]	Case–control	Netherlands	103 subfertile and 117 fertile men	34.3 ± 3.90	Seminal plasma	AAS	A significant positive correlation between seminal Zn level and sperm count. All data refer to cases.
Vashisht et al. (2021) [68]	Case–control	India	50 infertile (non-obstructive azoospermia, asthenozoospermia, normozoospermic infertile, and oligozoospermia and 50 fertile men	24–40	Seminal plasma	AAS	A significant positive correlation between seminal Zn level and sperm motility. All data refer to cases.
Chen et al. (2024 b) [79]	Case–control	China	374 normozoospermic men, 525 oligoasthenozoospermic, and 65 azoospermic men	19–66 (mean: 33 years)	Seminal plasma	Spectrophotometry	Significant positive correlations between seminal Zn^2+^ levels with sperm concentration and total sperm motility. Significant negative correlations between seminal Zn^2+^ levels with semen volume and Zn^2+^ concentration. All data refer to cases.
Jeng et al. (2015) [73]	Cross-sectional	Taiwan	196 men	38.4 ± 9.9	Seminal plasma, urine	AAS	No significant correlations between seminal or urine Zn levels with sperm quality. All data refer to cases.
Osadchuk et al. (2021) [80]	Cross-sectional	Russia	626 men	25.1 ± 7.40	Semen	Spectrophotometry	Significant positive correlations between seminal Zn levels with the total sperm count, sperm concentration, progressive motility, and normal morphology.
Chen et al. (2024 a) [75]	Cross-sectional	China	25,915 men	31.1 ± 5.19	Seminal plasma	Spectrophotometry	A significant positive correlation between seminal plasma Zn levels and sperm motility.

N.P. = not provided; AAS = Atomic Absorption Spectroscopy; TXRF = Total Reflection X-rays Fluorescence; ICP-MS = Inductively Coupled Plasma–Mass Spectrometry.

**Table 2:** Effects of Zn supplementation on blood/serum testosterone levels.

Reference	No.	Duration (Weeks)	Dose	Total Testosterone Levels (ng/dL) Before/After Treatment
Shafiei Neek et al. (2011) [88]	32	4	30 mg/day ZnSO_4_	~550/~750 *
Jalali et al. (2010) [86]	12 I/15 P	8	30 mg/day of Zn monomethionine aspartate + 450 mg of Mg aspartate + 10.5 mg of vitamin B6	567/752 *
Brilla and Conte (2000) [89]	7 I/7 P	8	30 mg/day of Zn monomethionine aspartate + 450 mg of Mg aspartate + 10.5 mg of vitamin B6	~650/~650
Koehler et al. (2009) [90]	100	6	250 mg of ZnSO_4_	447/847

I—intervention, P—placebo; * significant differences (*p* < 0.05).

## Data Availability

The data evaluated in this review are available from the corresponding author per reasonable request.
